# Cerebral/Cortical visual impairment (CVI) in Down syndrome: a case series

**DOI:** 10.3389/fnhum.2025.1563420

**Published:** 2025-07-11

**Authors:** Elizabeth Boatwright, Rudaina Banihani, Ilse Willems, Kathleen Lehman, Ellen Mazel, Hannah Mark, Mike Wong, Silvia Vietzman, Arvind Chandna, Gena Heidary, DSMIG DS+CVI Workgroup

**Affiliations:** ^1^Independent Internal Medicine-Pediatrics Physician, Educator, and Researcher, Colorado Springs, CO, United States; ^2^DAN Women & Babies Program, Department of Newborn & Developmental Pediatrics, Sunnybrook Health Sciences Centre, Toronto, ON, Canada; ^3^Department of Pediatrics, University of Toronto, Toronto, ON, Canada; ^4^CVI Center, Perkins School for the Blind, Educational Programs, Watertown, MA, United States; ^5^Institute on Human Development and Disability, University of Washington, Seattle, WA, United States; ^6^Department of Psychiatry & Behavioral Science, University of Washington School of Medicine, Seattle, WA, United States; ^7^Department of Social and Behavioral Sciences, Yale School of Public Health, New Haven, CT, United States; ^8^SeeLab, Smith-Kettlewell Eye Research Institute, San Francisco, CA, United States; ^9^Translational Clinical Eye Research Centre, Alder Hey Children’s Hospital, Liverpool, United Kingdom; ^10^Department of Ophthalmology, Boston Children’s Hospital and Harvard Medical School, Boston, MA, United States

**Keywords:** Cerebral/Cortical visual impairment (CVI), Down syndrome (DS)/Trisomy 21, Down syndrome and Cerebral/Cortical visual impairment (DS + CVI), visual attention, Top-11 Higher Visual Function Question Inventory (HVFQI), functional vision assessment (FVA), case report, visual perception

## Abstract

Cerebral/Cortical visual impairment (CVI), a brain-based visual condition, is a leading cause of childhood blindness and low vision but remains underdiagnosed in individuals with Down syndrome (DS). This report presents three case studies of adolescents with DS and CVI (DS + CVI), illustrating how CVI can manifest alongside the developmental, cognitive, behavioral, and social profiles of individuals with DS. We describe comprehensive ophthalmological evaluations, assessment for visual perceptual deficits with a screener questionnaire, functional vision assessments, and neuroimaging (when available). Through a detailed retrospective examination of these cases, we explore the complex interplay between CVI and DS, emphasizing how CVI-related challenges—such as difficulties with visual attention, spatial perception, processing, and navigation—are often misattributed to DS alone or to other more commonly recognized co-occurring conditions in DS. Diagnostic overshadowing, coupled with a lack of standardized screening tools, has led to delayed diagnoses and missed opportunities for intervention. Our findings highlight the importance of recognizing CVI in individuals with DS using reliable tools for assessment of functional vision to better appreciate the effect on their diverse developmental outcomes, and to incorporate CVI into our understanding of the DS phenotype. These case reports underscore the need for further research to determine the prevalence and impact of CVI in DS and advocate for the development of tailored screening protocols and evidence-based interventions to support individuals with DS + CVI across the lifespan.

## Introduction

Cerebral/Cortical visual impairment (CVI) is a brain-based visual impairment and the leading cause of childhood blindness and low vision in the United States (Chang et al., 2024), with a rising global prevalence (Teoh et al., 2021; Pehere et al., 2019). CVI is often linked to early brain injuries, including perinatal hypoxia, neonatal complications, and genetic conditions such as Down syndrome (DS) (Lehman et al., 2024; Pehere et al., 2018; Wilton et al., 2021). While CVI is increasingly recognized in other neurodevelopmental conditions, it remains under-identified in DS despite its potential impact on development (Wilton et al., 2021; Chokron and Dutton, 2016; Dale and Sonksen, 2002; Hatton et al., 1997; Mosca et al., 2015).

DS is a complex neurogenetic condition caused by the duplication of all or part of chromosome 21, making it the most common chromosomal anomaly (Mai et al., 2019). Developmental outcomes in DS vary widely (Frank and Esbensen, 2015; Winders et al., 2018), shaped by a dynamic interplay of co-occurring medical (Bull et al., 2022; Baksh et al., 2023; Amr, 2018; Breslin et al., 2014; Laws and Hall, 2014; Rako et al., 2021), ophthalmologic (Jain et al., 2023), and neurodevelopmental conditions, including autism spectrum disorder (ASD) (Richards et al., 2015; Fidler et al., 2022) and attention-deficit/hyperactivity disorder (ADHD) (Ekstein et al., 2011). Advances in research have deepened our understanding of these conditions (Antonarakis et al., 2020), and evidence-based care guidelines have improved life expectancy and quality of life (Bull et al., 2022; Tsou et al., 2020). Our understanding of the strengths and challenges in DS continues to evolve (Fidler, 2005; Schworer et al., 2022). Recognizing variability in medical, behavioral, social, and cognitive profiles is crucial for developing tailored interventions (Fidler and Nadel, 2007; Onnivello et al., 2023a). Despite improvements in care, individuals with DS exhibit a broad range of developmental and cognitive trajectories (Baumer et al., 2024; Channell et al., 2021), suggesting that additional unrecognized factors contribute to these differences.

We propose that CVI is one such overlooked factor. Currently, CVI is absent from DS screening guidelines despite its potential to significantly affect developmental, cognitive, and social outcomes.

This retrospective case report examines three adolescent females with DS and CVI (DS + CVI), analyzing long-term medical, developmental, and behavioral data. Clinical records were reviewed, including ophthalmological evaluations, neuroimaging (when available), structured screening tools for visual perceptual deficits, and functional vision assessments. We reconstruct each diagnostic trajectory, emphasizing early neonatal, medical, and developmental factors contributing to delayed CVI recognition. We highlight diagnostic overshadowing, where CVI-related difficulties—such as impaired visual attention, processing, and navigation—were misattributed to DS or other co-occurring conditions. By analyzing these cases, we illustrate how CVI manifests in DS and advocate for systematic CVI screening and integration into routine clinical care to improve developmental outcomes.

## Case reports

### Case 1

Born at term, this female infant had APGAR scores of 8 and 8 at one and 5 min. She required oxygen supplementation for mild respiratory distress during a 10-day stay in the neonatal intensive care unit (NICU), during which time she was treated briefly with phototherapy for transient hyperbilirubinemia. A hemodynamically stable patent ductus arteriosus (PDA) was identified but resolved spontaneously. No significant perinatal complications were recorded (Table 1).

**Table 1:** Medical history.

Medical issue	Case 1	Case 2	Case 3
Pregnancy	Uneventful, no prenatal diagnosis	Prenatal diagnosis of DS and VSD	Complicated with oligohydramnios, prenatal diagnosis of DS and CAVC
Delivery	NSVD, APGARS 8/8	NSVD, APGARS 8/9	C/S, APGARS unknown
Gestational age (GA)	41 weeks	39 weeks	36.5 weeks
Birth weight—SGA (<3rd percentile)	No	No	No
Congenital heart disease (CHD)	PDA (no surgery)	VSD (no surgery)	CAVC (surgical repair age 2 months, uneventful)
PPHN	Present	None	None
Evidence of NE	None	None	None
Neonatal hyperbilirubinemia	Yes, phototherapy 2 days	Yes, phototherapy 10 days	None
Complex eye conditions	Yes	Yes	Yes
Hearing Problems	Fluctuating conductive hearing loss	Conductive & sensorineural hearing loss	Fluctuating conductive hearing loss
	Eustachian tube dysfunction, PE tubes	Eustachian tube dysfunction, PE tubes	Eustachian tube dysfunction, PE tubes
	No augmentative devices	Bilateral hearing aids	FM system at school
Thyroid disease	None	Hyperthyroid	None
Hip instability	Yes, treated with PT	No	Yes, multiple surgeries

PPHN, persistent pulmonary hypertension of the newborn; NE, neonatal encephalopathy; CAVC, complete atrial-ventricular canal; VSD, ventricular septal defect; PDA, patent ductus arteriosus; FM, frequency modulation; PE, pressure equalizing.

**Figure 1:**
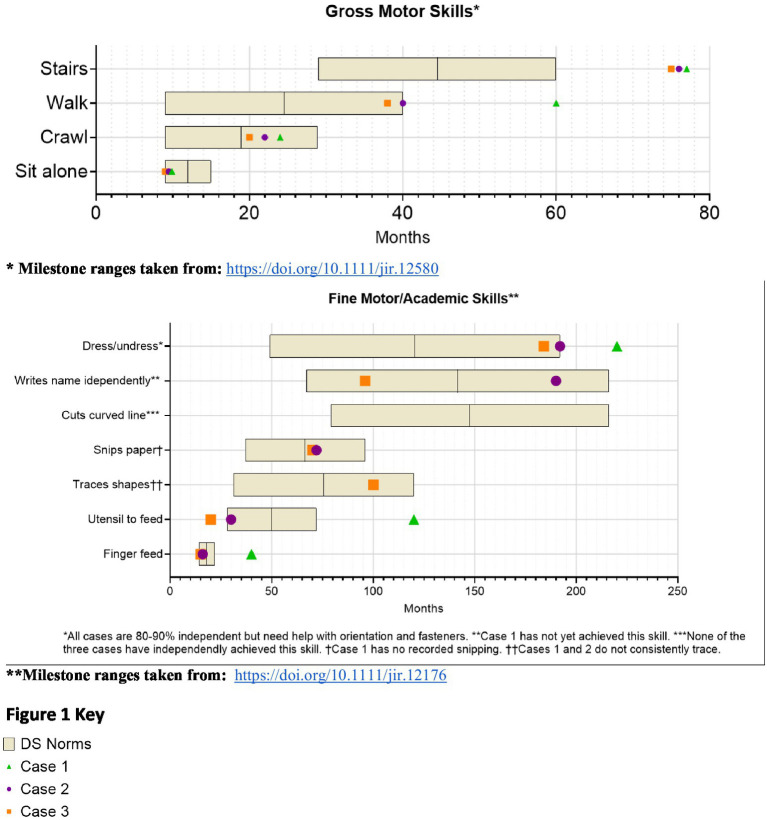
Developmental trajectories.

From infancy, delays in visual-motor integration were apparent, and inconsistent reaching behaviors and limited eye-hand coordination persisted; at age 3 year, she was not using hands and eyes together. Gross motor skills showed progressive delays (Figure 1). Hip instability contributed to persistent difficulty with gait. Early assessments confirmed delays across cognitive, language, and motor domains, with persistent fine motor challenges such as difficulty reaching, holding objects or writing. Socially, she engaged well in near-gaze interactions but displayed variable joint attention. Language development slowed, particularly after stopping sign language, but she does rely primarily on spoken language, often using scripted phrases and echolalia. At age 14, she underwent a multidisciplinary ASD evaluation and was diagnosed with autism (Table 2).

**Table 2:** Summary of Developmental Diagnoses and Standardized Assessments.

Case	ASD Diagnosis*	ASD Diagnostic Tools Employed	ADHD Diagnosis*	ADHD Diagnostic Tools Employed
1	Yes	GARS-3; ASRS; ADOS-2	Not assessed	N/A
2	No	ADOS-2	Not assessed	N/A
3	Not assessed	N/A	Yes	VADRS, Parent and Teacher Forms

* Diagnoses were made based on DSM-5 criteria through multidisciplinary team assessment.

ASD, Autism Spectrum Disorder; ADHD, Attention Deficit Hyperactivity Disorder; ADOS-2, Autism Diagnostic Observation Schedule, Second Edition; ASRS, Autism Spectrum Rating Scales; DSM-5, Diagnostic and Statistical Manual of Mental Disorders, Fifth Edition; GARS-3, Gilliam Autism Rating Scale, Third Edition; VADRS, Vanderbilt ADHD Diagnostic Rating Scale; N/A, Not Applicable.

Ophthalmologic evaluations revealed significant nystagmus by 2 months (Table 3A). She has been treated for strabismus with surgery at age 8 and though she wears corrective lenses for her high myopia, her most recent best-corrected binocular visual acuity (VA) is limited at 20/80 (monocular testing was difficult). She was diagnosed with CVI at 4 months due to lack of visual fixation not explained by ocular findings. She received vision services through school, but her parents and school were led to believe that her CVI had resolved by middle school. Reevaluation at age 19 using the TeachCVI Screener #2 (TeachCVI Project, 2017; Martin et al., 2024) was prompted by ongoing functional vision concerns and confirmed persistent CVI-related deficits (Table 3B). While MRI was not performed, functional assessments revealed significant challenges with clutter, motion, and depth perception. Behavioral patterns such as mirror aversion, difficulty visually following another’s pointing finger to distant objects, and sensitivity to loud sounds became more pronounced by adolescence.

**Table 3:** Visual function and functional vision.

(A) Ophthalmologic history and evaluation			
Eye exam, ophthalmologic conditions	Case 1	Case 2	Case 3
Glasses Rx	−8.00 + 3.25 × 65 OD, −7.25 + 3.00×77 OS	+3.75 + 2.50 × 82 OD, +4.25 + 2.50 × 104 OS	−2.50 + 2.00 × 015 OD, +0.75 sphere OS
Best corrected binocular acuity	20/80	20/50	20/30
Strabismus	+Esotropia surgery age 8 years	+Esotropia managed with bifocals	+Exotropia surgery age 7.5 years
Nystagmus	Present	Absent	Absent
Cataracts	+Mild (not visually significant)	None	None
Abnormality of retina or optic nerve	None	None	None
CVI diagnosis	Age 4 months	Age 15.5 years	Age 15 years

(B) Select CVI visual behaviors evaluated by functional visual assessments (FVA)			
Visual behavior	Case 1, age 19 years^*^	Case 2, age 15.5 years^**^	Case 3, age 15 years^**^
Visual attention	Brief glances	Brief glances	Brief glances
Visual recognition	Recognizes a set of familiar simple 3D objects; 2D is mostly inaccessible	Recognizes familiar and simple 3D; can recognize certain simple colored photographs	Recognizes familiar 3D objects and realistic colored images and photographs. Recognizes sight words by overall shape of the word
Impact of clutter and crowding	Clutter interferes with visual attention, finding objects	Clutter interferes with visual attention, finding objects; best with 1–2 objects at a time	Clutter interferes with visual attention, finding objects; best with 1–2 objects at a time
Visual field abilities	Lowered head, lowering accessible field	Decreased right, upper fields	Right stronger than left field, decreased use of lower field
Impact of color	Relies on color coding to recognize	Relies on color coding to recognize; drawn to bright colors	Relies on color coding to recognize; drawn to bright colors
Visual guidance of upper limbs	Inconsistently looks while reaching	Inconsistently looks while reaching	Inconsistently looks while reaching
Access to people	Brief eye contact	Uses visual and auditory cues to identify people	Brief eye contact; misidentifies facial expressions
Impact of light	Frequently stares at light sources; backlighting supports visual attention; sensitive to glare	Frequently stares at light sources; prefers low ambient light and task lighting; very sensitive to glare and bright light (can trigger migraine)	Sometimes stares at light sources; backlighting supports visual attention; sensitive to glare (can trigger migraine)
Impact of motion	Motion in the environment can be distracting and overwhelming; small movements draw attention	Motion in the environment can be distracting and overwhelming; often sits in busy environments; small movements draw attention	Motion in the environment can be distracting and overwhelming; small movements of cursor draws attention
Sensory integration and impact on vision	Difficulty looking and listening simultaneously; noises in the environment can be startling and upsetting	Difficulty looking and listening simultaneously; noises in the environment can be startling and upsetting	Difficulty looking at an item she’s holding; noises in the environment can be startling and upsetting
Support of compensatory skills	Benefits from auditory and verbal cues, color coding, prediction, and memory	Benefits from auditory cues, color coding, and tactile cues	Benefits from auditory and verbal cues, color coding, and tactile skills
^*^Assessed with: https://www.teachcvi.net/screening-tools			
^**^Assessed with: https://www.perkins.org/our-work/cvi/the-perkins-cvi-protocol/			

(C) Comparison of higher order visual function screening results: top-11 questions^*^			
Top-11 questions	Case 1	Case 2	Case 3
1. Does your child sometimes have difficulty seeing something that is pointed out in the distance?	5	5	4
2. Does your child find uneven ground difficult to walk over?	5	5	5
3. Does your child bump into things when walking and having a conversation?	1 (very cautious, stops talking to walk)	3 (usually does not walk & talk)	5
4. Does your child have difficulty walking downstairs?	3	5	5
5. After being distracted does your child find it hard to get back to what they were doing?	3	4	4
6. Does your child have difficulty finding a close friend or relative who is standing in a group	2	4	5
7. Does your child find copying words or drawings time-consuming and difficult	5	5	4
8. Does your child trip at the edges of pavements going down?	2	2 (stops)	3
9. Does your child find inside floor boundaries difficult to cross?	2	4	5
10. Does your child have difficulty seeing scenery from a moving vehicle?	2	5	4
11. Does your child look down when crossing floor boundaries?	5	5	5
^*^Doi: 10.3389/fnhum.2021.711873			

Likert scoring rubric (note adjusted scores compared to 2021 paper).

Questions answered by parents have a choice of answers with scores: never—score 1; rarely—score 2; sometimes—score 3; often—score 4; always—score 5.

Presently, focused CVI accommodations, including the use of contrasting backgrounds, color coded visual schedules, and clutter reduction, have enhanced her ability to navigate both academic and social environments. Behavioral interventions emphasizing multisensory learning have further supported her development, although she struggles to process information from visual, auditory, and motor channels at the same time. Academic skills are minimal and life skills have been emphasized at school.

### Case 2

This female infant was born at term after a pregnancy complicated by a prenatal diagnosis of ventricular septal defect (VSD), with APGARS of 8 and 9 at one and 5 min. Her neonatal course included transient tachypnea of the newborn (TTN) and mild hyperbilirubinemia, requiring a 7-day NICU stay for observation and treatment. She stabilized quickly and was discharged without complications to continue phototherapy as an outpatient for a total of 10 days (Table 1).

Developmental milestones revealed early gross motor skills in normal limits for DS, with progressive delays (Figure 1). Visuomotor challenges became evident during infancy, with limited reaching and grasping. While she initially learned baby signs, verbal language development slowed after sign language use was discontinued, leading to delays in communication noted by age 3. Early assessments confirmed global developmental delays. Socially, she was interactive but struggled with joint attention and sustained eye contact. She later displayed strong phonics-based reading skills but struggled to read multiple words on a page and had difficulty tracing and writing. Additional medical conditions include mild hearing loss, hyperthyroidism, obstructive sleep apnea, and migraines (Table 1). By age 10, concerns about social behavior and repetitive movements led to an ASD evaluation, which ruled out the diagnosis (Table 2).

Ophthalmologic findings included hyperopia (Table 3A). Esotropia was managed with bifocals. At the most recent follow up, best corrected binocular VA is 20/50 (monocular testing was difficult). Behavioral observations highlighted difficulty navigating stairs and hesitating at thresholds, and heightened sensitivity to sound and motion which interfered with group activities and transitions.

Persistent functional vision concerns prompted CVI evaluation at age 15.5. Functional visual assessments using The Perkins CVI Protocol (CVI Center at Perkins School for the Blind, 2024) confirmed significant deficits in visual attention, clutter, motion, glare, and depth perception (Table 3B). Brain MRI showed increased susceptibility in the basal ganglia regions (Table 4).

**Table 4:** Imaging and EEG findings.

Diagnostic tools	Case 1	Case 2	Case 3
MRI findings	Not performed	Mildly small brainstem Increased susceptibility in globus pallidus and substantia nigra	Pons hypoplastic, mildly hypoplastic inferior vermis Increased susceptibility in globus pallidus R > L
EEG findings	Not performed	Diffuse slowing, no epileptic foci	Diffuse encephalopathy, nonspecific etiology

Currently, CVI-specific interventions, such as the use of slant boards, dimmed overhead lighting with task lighting, realistic photographs, a 3D schedule system, and multisensory learning aids have improved her academic engagement. Structured routines, deaf-blind supports, and cane use have reduced her anxiety and improved her participation in group settings.

### Case 3

Born preterm at 36.5 weeks via cesarean section due to decreased amniotic fluid, this female infant was vigorous at birth (APGARS unknown). Prenatal imaging identified a complete atrioventricular canal (CAVC), which required surgical repair at 2 months. Her neonatal course was otherwise uneventful, with a brief 2-day NICU stay for monitoring.

From infancy, developmental delays were apparent, particularly in visuomotor skills. Developmental milestones revealed early gross motor skills in normal limits for DS, with progressive delays (Figure 1). Language development progressed steadily due to consistent use of multimodal communication methods. Early assessment confirmed significant cognitive, motor, and language delays. Socially, she demonstrated a preference for humor and verbal engagement, often incorporating lines from songs or movies into conversations. Additional medical conditions included congenital heart disease requiring repair, hip instability requiring surgery, obstructive sleep apnea, fluctuating hearing loss, and migraines. Behavioral challenges included difficulty navigating crowded environments, difficulty with transitions, and noncompliance and aggressive behavior in a busy classroom. She was asked to leave her inclusive school setting after 4th grade, after aggressive behaviors including hitting, yelling, and pushing over tables accelerated. At age 13, she was diagnosed with ADHD, which improved with pharmacological treatment.

Ophthalmologic evaluations revealed exotropia with significant myopic astigmatism OD and mild hyperopia OS indicating significant anisometropia but no amblyopia. Exotropia was surgically corrected at age 7.5. At the most recent follow up, her best corrected binocular VA is 20/30 (20/40 monocular VA).

CVI evaluation at age 15 confirmed significant visual processing deficits, including clutter and navigation difficulties. Brain MRI findings indicated increased susceptibility in the globus pallidus, and functional assessments corroborated CVI-related impairments.

Today, CVI-specific accommodations, including the use of backlighting, clutter-free environments, realistic photographs, and cane use have improved her ability to navigate both academic and social contexts.

### Diagnostic assessments across the three cases

The diagnosis of CVI in the three cases was established through a systematic and multidisciplinary approach, incorporating birth history, comprehensive eye examinations, assessment with semi-structured question inventory screener, functional vision assessments, and neuroimaging findings where available (see discussion in Pilling et al., 2023). We used the validated Top-11 CVI Screener and HFVQI-51 semi-structured interview (Chandna et al., 2024), completed by parents of the cases, to assess higher visual function deficits. Table 3 provides a comparative analysis of CVI-related behaviors across the cases, illustrating distinct yet overlapping profiles.

Diagnostic assessments revealed a shared constellation of challenges consistent with CVI (Fazzi et al., 2007; Boot et al., 2010; Philip and Dutton, 2014; Sakki et al., 2018), including visual perceptual deficits and visually guided motor behavior delays, leading to challenges with functional vision. All three cases showed both visual function deficits (decreased VA, decreased visual fields, oculomotor impairment), as well as functional vision deficits of the ventral stream (e.g., route finding and orientation, recognition) and dorsal stream (e.g., visually guided movement, motion perception) (Chang et al., 2024). All three had difficulty integrating sensory information (auditory, visual, tactile at one time), to varying degrees. This sensory sensitivity occasionally led to episodes of visual overwhelm, manifesting as shutdowns or vasovagal reactions, sometimes triggering migraines. Compensatory behaviors, such as trailing hands along walls or hesitating before navigating unfamiliar spaces, reflected attempts to adapt to a visually unpredictable world.

Notably, all three cases scored in the high-severity and spectrum of CVI, exceeding normative thresholds established for neurotypical individuals and aligning with findings from other CVI cohorts without DS. The mean severity scores for each case were 2.4, 3.27, and 3.45 (numerical scale: never = 1; rarely, sometimes, often, always = 5) and significantly higher than the mean severity score from normative data (see below). Because of high severity scores on the Top-11 screener, all three cases were evaluated with the detailed HVFQI-51, a structured 51 question inventory (Chandna et al., 2021). Severity scores (SD) for HVFQI-51 for the three cases were: 3.39 (0.18); 3.05 (0.77); 3.04 (0.49), values considerably different from the normative cohort (1.43 S.D. 0.49 cohort *n* = 120) (Chandna et al., 2024).

Although there were similarities in functional visual profiles, the three cases had unique variations and varying levels of severity. These findings align with prior reports emphasizing the heterogeneity of CVI-related sensitivities (Philip and Dutton, 2014).

Neuroimaging findings by MRI scans did not show clear lesions in afferent visual pathways, but did reveal differences in basal ganglia structures, specifically the globus pallidus and substantia nigra. EEG findings in both cases indicated nonspecific slowing.

## Discussion

The findings across the three cases emphasize the intricate interplay of developmental, behavioral, and functional challenges associated with CVI in individuals with DS. Each of the three cases had progressive developmental delays compared to DS norms, as visualized in Figure 1, yet each case had a unique developmental trajectory, underscoring the individualized effect of CVI in DS amidst the varied factors influencing overall development. These cases demonstrate the confusing overlap between DS and CVI, ASD, and ADHD (Chokron and Dutton, 2023), as well as the challenge of distinguishing ASD in VI (Molinaro et al., 2020). Functionally, challenges with visual perception and visually guided motor behavior impacted activities of daily living, navigation, leisure activities, and independence. Because of diagnostic overshadowing and lack of awareness of CVI, the complex challenges faced by the three cases were easily attributed to DS alone, concurrent medical conditions, or co-occurring neurodevelopmental conditions like ASD and ADHD, highlighting current challenges with diagnosing CVI in DS.

Clear vision is fundamental to achieving optimal developmental and functional outcomes in individuals with DS, particularly given their strengths as social learners and visual processors (Rosenbaum et al., 2024; Rosenbaum and Gorter, 2012). A heightened awareness of DS + CVI is important as early identification of CVI in DS may lead to early intervention and individualized accommodations that might improve the trajectory of visual development and potentially impact overall development, fostering greater independence and well-being for individuals with DS + CVI across their lifespan.

All three cases had complex ocular conditions common in DS (Jain et al., 2023) and were followed by ophthalmology from birth, but CVI was missed in two cases until the teen years, due to lack of awareness, as well as the lack of clearly defined CVI risk factors, screening guidelines, and diagnostic criteria that would prompt a CVI evaluation in DS. These cases share some common elements in their birth histories, clinical ophthalmologic exams, and imaging studies that may serve as a basis for further study of factors increasing vulnerability to CVI in DS.

One key observation is that none of the cases had classic risk factors for CVI at birth (Chang et al., 2024), but all three birth histories included medical events common for babies with DS, such as brief NICU stays, oxygen supplementation, and treatment for hyperbilirubinemia. While these medical factors are not traditionally recognized as CVI risk factors, emerging evidence outside the DS population suggests that even mild hypoxia (James et al., 2019; Nagy et al., 2021; Li et al., 2022) and mild hyperbilirubinemia in term infants (Amin et al., 2019; Hou et al., 2014) may have subtle but lasting effects on visual pathways in the brain, and both are common in neonates with DS.

In addition, these cases had clinical ophthalmologic findings of subnormal visual acuity (VA) and visual perceptual deficits that persisted despite traditional corrective measures of their complex ocular conditions like strabismus and nystagmus. Complex ocular conditions are common in DS (Santoro et al., 2024) but also occur in CVI (Fazzi et al., 2007), and the fact that the complicated visual profiles of these cases did not fully account for the observed functional vision deficits in these cases is a hallmark feature of brain-based visual impairment. Subnormal VA is common in DS, and may serve as a clinical finding that should prompt further questioning and evaluation for CVI.

MRI findings in two cases align with established neuroimaging patterns in DS alone, and include basal ganglia anomalies, which are more prevalent in DS than in the general population (Santoro et al., 2024; Takashima and Becker, 1985; Lee et al., 2013). While linked to depth perception, motor coordination, and contextual learning challenges in non-DS populations (Milardi et al., 2019; Paprocka et al., 2020), their functional significance in DS remains unclear.

These cases suggest that a CVI diagnosis benefits individuals with DS + CVI, their families, and their care teams. According to parents, the CVI diagnosis validated their concerns about their teen’s vision, provided them with a new framework for understanding their teens’ behavior, increased their empathy and patience, and provided new tools to support their loved ones. Anecdotally, therapeutic interventions targeting each teen’s CVI-specific needs have improved functionality, reduced anxiety, and increased engagement in learning environments in all three cases. However, further research on CVI accommodations would help clarify the effectiveness of interventions.

Despite its retrospective nature (reliance on parental recall for early developmental milestones and behaviors, incompleteness of records, and variability of diagnostic tools), this case report provides compelling evidence for further research into CVI’s prevalence, spectrum, and impact within the DS population. The cases presented are teenagers, which may limit the generalizability of findings to younger children, and a case study by nature will not capture the breadth of presentations of CVI in DS.

However, together with recent publications describing nuanced profiles of development in DS (Onnivello et al., 2023a; Onnivello et al., 2023b), cognition in DS (Channell et al., 2021) and autism symptoms in DS (Fidler et al., 2022), these cases raise the possibility that CVI and ocular visual impairments may influence the broader DS phenotype in ways previously unrecognized. To help clarify the role of visual function deficits in DS, prospective studies of development in DS which consider the impact of vision and studies that identify and quantify higher visual function deficits in the larger DS population are necessary. A detailed multidisciplinary assessment of ASD and/or ADHD-like challenges in DS, particularly in familiar versus novel environments, is essential to accurately differentiate between vision-related behaviors and those associated with the neurodevelopmental disorders. Modifications to existing diagnostic tools to account for visual impairments may improve accuracy in these populations (Williams et al., 2014). Incorporating CVI assessments within the already established ophthalmology screening protocols recommended by the AAP may improve early identification, facilitate timely interventions, and enhance developmental outcomes.

Future research should focus on refining CVI-specific screening tools tailored to the DS population and validating interventions for CVI and the long-term impact of CVI accommodations on functional outcomes and quality of life. Additionally, conducting cohort studies involving individuals with DS both with and without CVI, using standardized CVI semi-structured questionnaire interviews such as the Top-11 CVI Screener and HFVQI-51 could provide critical insights into the prevalence, spectrum and severity of HVFDs in this population. These studies should include neuroimaging analyses, including functional MRI (fMRI) (Hirsch et al., 2015), to identify potential biomarkers, anatomical, and neurological differences that may contribute to visual processing challenges, alongside looking at early neonatal, medical, and developmental characteristics contributing to CVI + DS versus DS alone.

## Data Availability

The datasets presented in this article are not readily available because they are the property of SeeLab, Smith-Kettlewell Research Institute. Permission must be granted by AC, via the corresponding author. Requests to access the datasets should be directed to arvind.chandna@alderhey.nhs.uk.

## Group members of DSMIG DS+CVI Workgroup

Elizabeth Boatwright, Independent Internal Medicine-Pediatrics Physician, Educator, and Researcher, Colorado Springs, CO, United States; Rudaina Banihani, DAN Women & Babies Program, Department of Newborn & Developmental Pediatrics, Sunnybrook Health Sciences Centre, Toronto, ON, Canada, Department of Pediatrics, University of Toronto, Toronto, ON, Canada; Ilse Willems, CVI Center, Perkins School for the Blind, Watertown, MA, United States; Kathleen Lehman, Institute on Human Development and Disability, University of Washington, Seattle, WA, United States, Department of Psychiatry & Behavioral Science, University of Washington School of Medicine, Seattle, WA, United States; Arvind Chandna, SeeLab, Smith-Kettlewell Eye Research Institute, San Francisco, CA, United States, Translational Clinical Eye Research Centre, Alder Hey Children’s Hospital, Liverpool, United Kingdom; Gena Heidary, Department of Ophthalmology, Boston Children’s Hospital and Harvard Medical School, Boston, MA, United States.
